# To determine whether the whole recovery‐oriented system of care is greater than the sum of its parts, we must start by describing the parts

**DOI:** 10.1111/add.70102

**Published:** 2025-06-05

**Authors:** Ed Day, Suzie Roscoe, Laura Pechey, John Kelly

**Affiliations:** ^1^ Institute for Mental Health, School of Psychology University of Birmingham Birmingham UK; ^2^ Department of Health and Social Care Office for Health Improvement and Disparities London UK; ^3^ Harvard Medical School and Center for Addiction Medicine, Recovery Research Institute Massachusetts General Hospital Boston MA USA

**Keywords:** alcohol, collegiate recovery program, drugs, employment support, peer‐based recovery support services, recovery check‐up, recovery coaching, recovery community centre, recovery housing

*Breaking down the elements of the recovery oriented system of care (ROSC) for addiction has been helpful to describe key interventions and their supporting evidence‐base. However, integration of these different elements is crucial, and alternative theory‐based research and evaluation paradigms may help understand the underlying processes and their likely outcomes when introduced in a variety of contexts*.

Our scoping review of recovery support services (RSS) for addiction [1] was conducted to inform the development of policy guidance for commissioners of government‐funded treatment and recovery services in England [2]. It represented a very practical attempt to apply ideas of recovery, peer support and continuing care to policymaking in the treatment and recovery space. By delineating specific RSSs and describing their evidence‐base and connection to the wider continuum of care we hoped to facilitate communication around key elements of recovery support. The commentaries on the monograph reflect on our findings with reference to three separate national systems. Ivers [3] considers how the monograph can inform the development of a similar national strategy in Ireland. As in the United Kingdom, she feels that this process reflects ‘the growing consensus that recovery is a dynamic, lifelong process rather than a finite clinical outcome’, and an interest in using ‘coordinated networks of clinical and non‐clinical services…to improve recovery outcomes.’ In Belgium, Vanderplaschen and colleagues [4] report that some RSS elements have been implemented (peer‐based services and continuing care), but others are still awaited. Finally, Samion *et al*. [5] identify multiple strategies for building recovery capital in service provision in a different cultural context in Singapore, including continuing care, employment support, peer‐based recovery support services (PBRSS) and recovery housing.

The commentators reflect on both the utility and the limitations of our approach, and two points are worth making. First, there is a need to tailor these ideas to the unique social and cultural context in which they are being applied. The commentators note the overwhelming focus on research published in English in predominantly White, ‘Anglo‐Saxon’ cultures, when other alternative models of recovery support exist [4]. While this focus was intentional because of shared cultures and behaviours, learning can be taken from less similar populations. For example, indigenous populations of Canada and New Zealand both emphasise the importance of human connection and community. Aboriginal belief systems in Canada place the emphasis on the interconnection of all aspects of well‐being (including physical, emotional, mental and spiritual), the adoption of a lifespan approach and the understanding of individual health as an aspect of the health of families, communities, nations and the environment [6]. Likewise, mental health policy in Aotearoa New Zealand has recognized the extended family (whānau ora) as a source of strength, identity and support [7].

Second, although the separate RSS elements make sense, there is consensus that when these elements operate independently their effectiveness is constrained. Integration of the different components into the treatment and recovery system is crucial, as well as integration into other systems of care such as mental health. General population surveys in both the United States [8] and the United Kingdom [9] remind us that a majority of people recover without treatment or formal support of any kind. However, those with complex problems may need structured support, particularly as mortality is high without it [10]. Our scoping review starts the process of how best to visualise this support, how to structure it, introduce it, monitor it and evaluate it. However, the key question is evaluating how these various elements work together as part of a recovery oriented system of care (ROSC). Breaking the ROSC down into individual RSS components allowed the emphasis to be on experimental and quasi‐experimental impact evaluation methods. However, in practice most RSS comprise several components delivered in one or more setting types. Another approach may be to explore the causal chains thought to bring about change through the implementation of a fully realised ROSC. Such theory‐based approaches, including realist evaluation, are explicitly concerned with the extent of the change, why the change occurs and the context in which it happens [11]. Co‐producing this approach with people with lived experience of addiction may be the best way to understand the most effective implementation strategies and the best outcomes to measure when evaluating success.

## AUTHOR CONTRIBUTIONS

**Ed Day:** Writing—original draft; writing—review and editing. **Suzie Roscoe:** Writing—review and editing. **Laura Pechey:** Writing—review and editing. **John Kelly:** Writing—review and editing.

## DECLARATION OF INTERESTS

None.

## References

[1] Day E , Pechey LC , Roscoe S , Kelly JF . Recovery support services as part of the continuum of care for alcohol or drug use disorders. Addiction. 2025;120(8):1497–1520. 10.1111/add.16751 39873444 PMC12215296

[2] Pechey L , Roscoe S , Day E . Recovery support services and lived experience initiatives HM Government: Office for Health Improvement and Disparities. 2023.

[3] Ivers J‐HH . Commentary on Day et al.: Systemic solutions for recovery support services in Ireland's National Drug Strategy. Addiction. 2025;120(8):1524–1525. 10.1111/add.70029 40013365 PMC12215228

[4] Van der Plasschen W , De Meyer F , De Ruysscher C . Commentary on Day et al.: From concept to practice—Challenges in building a continuum of recovery support services in Belgium. Addiction. 2025;120(8):1521–1523. 10.1111/add.70020 40045750

[5] Samion S , Kaur J , Leung CC . Commentary on Day et al.: Singapore's approach toward drug rehabilitation. Addiction. 2025;120(8):1526–1528. 10.1111/add.70063 40183151

[6] Chansonneuve D . Addictive behaviours among Aboriginal people in Canada. In: The Aboriginal Healing Foundation research Ottawa, Canada: Aboriginal Healing Foundation; 2007.

[7] O'Hagan M , Reynolds P , Smith C . Recovery in New Zealand: an evolving concept? Int Rev Psychiatry. 2012;24(1):56–63. 10.3109/09540261.2011.651447 22385427

[8] Kelly JF , Bergman B , Hoeppner BB , Vilsaint C , White WL . Prevalence and pathways of recovery from drug and alcohol problems in the United States population: implications for practice, research, and policy. Drug Alcohol Depend. 2017;181:162–169. 10.1016/j.drugalcdep.2017.09.028 29055821 PMC6076174

[9] Day E , Manitsa I , Farley A , Kelly JF . The UK national recovery survey: A nationally representative survey of overcoming a drug or alcohol problem. BJPsych Open. 2024;10:e67. 38482691 10.1192/bjo.2023.654 PMC10951842

[10] Office for National Statistics . Deaths related to drug poisoning in England and Wales: 2023 registrations ONS; 2024.

[11] HM Treasury . The magenta book: Central government guidance on evaluation London: Crown Copyright; 2020.

